# Spectrum of Germline *BRCA1* and *BRCA2* Variants Identified in 2351 Ovarian and Breast Cancer Patients Referring to a Reference Cancer Hospital of Rome

**DOI:** 10.3390/cancers12051286

**Published:** 2020-05-19

**Authors:** Concetta Santonocito, Roberta Rizza, Ida Paris, Laura De Marchis, Carmela Paolillo, Giordana Tiberi, Giovanni Scambia, Ettore Capoluongo

**Affiliations:** 1 Molecular and Genomic Diagnostics Laboratory, Fondazione Policlinico Universitario A. Gemelli IRCCS, 00168 Rome, Italy; concetta.santonocito@unicatt.it (C.S.); robertarizza@gmail.com (R.R.); 2 Department of Biochemistry Catholic University of Rome, 00168 Rome, Italy; 3 Division of Gynecologic Oncology, Department of Woman and Child Health and Public Health, Fondazione Policlinico Universitario A. Gemelli IRCCS, 00168 Rome, Italy; ida.paris@policlinicogemelli.it (I.P.); giovanni.scambia@policlinicogemelli.it (G.S.); 4 Division of Medical Oncology A, Policlinico Umberto I, Sapienza University of Rome, 00168 Rome, Italy; laura.demarchis@uniroma1.it (L.D.M.); giordana.tiberi@libero.it (G.T.); 5 Department of Clinical and Experimental Medicine, University of Foggia, 71121 Foggia, Italy; Carmela.paolillo@gmail.com; 6 Dipartimento di Medicina Molecolare e Biotecnologie Mediche, Università Federico II, 80131 Naples, Italy; 7 CEINGE, Advanced Biotechnology, 80131 Naples, Italy

**Keywords:** next-generation sequencing, novel variants, *BRCA1/2*

## Abstract

Pathogenic variants (PVs) carriers in *BRCA1* or *BRCA2* are associated with an elevated lifetime risk of developing breast cancer (BC) and/or ovarian cancer (OC). The prevalence of *BRCA1* and *BRCA2* germline alterations is extremely variable among different ethnic groups. Particularly, the rate of variants in Italian BC and/or OC families is rather controversial and ranges from 8% to 37%, according to different reports. By In Vitro Diagnostic (IVD) next generation sequencing (NGS)-based pipelines, we routinely screened thousands of patients with either sporadic or cancer family history. By NGS, we identified new PVs and some variants of uncertain significance (VUS) which were also evaluated in silico using dedicated tools. We report in detail data regarding *BRCA1/2* variants identified in 517 out of 2351 BC and OC patients. The aim of this study was to report the incidence and spectrum of *BRCA1/2* variants observed in BC and/or OC patients, tested in at Policlinico Gemelli Foundation Hospital, the origin of which is mainly from Central and Southern Italy. This study provides an overview of the variant frequency in these geographic areas of Italy and provides data that could be used in the clinical management of patients.

## 1. Introduction

The *BRCA1* and *BRCA2* are two genes involved in double-strand DNA breaks repair by the homologous recombination system (HR). Pathogenic variants (PVs) in one of these genes, causing the absence or dysfunction of the BRCA proteins, can dramatically impair HR resulting in genomic instability. These PVs are deleterious and therefore increase an individual’s likelihood of developing cancer [1,2,3,4]. Deleterious germline PV carriers in *BRCA1* or *BRCA2* have an elevated lifetime risk of developing breast and/or ovarian cancer, particularly 60–80% for breast cancer (BC) and 26–54% for *BRCA1* and 10–23% for *BRCA2* for ovarian cancer (OC) [5,6]. In carrier men, the risk of developing BC is 1% and 6% for *BRCA1* and *BRCA2* PVs, respectively. PVs in these genes can also be involved in a higher risk of developing prostate cancer [7] and pancreatic cancer [8]. Genetic analysis of *BRCA1/2* genes identified more than 20,000 unique variants including missense, nonsense, frameshift, and site splicing variants as well as large rearrangements. The variants are classified and interpreted according to both the ACMG (American College of Medical Genetics) indications [9] and the ENIGMA (evidence-based network for the interpretation of germline mutant alleles) using the five-class system [10,11]. The prevalence of *BRCA1* and *BRCA2* germline variants is extremely variable among different ethnic groups. In particular, the rate of variants in Italian BC and/or OC families is rather controversial and ranges from 8% to 37%, according to different reports [12,13,14,15,16,17,18,19]. Apart from two founder variants recurring in individuals from Sardinia and Calabria [20,21], *BRCA1/2* variants are distributed throughout the entire coding sequence of the two genes. Use of next-generation sequencing (NGS)-based technologies allowed the screening of thousands of affected individuals, selected according to the young age at diagnosis or cancer family history. The knowledge of BRCA status in individuals with BC and/or OC can help in choosing treatment, especially for OC [20] and carry out cost-effective screening in first-degree relatives. The aim of this study was to report the incidence and spectrum of *BRCA1/2* variants observed in BC and/or OC patients tested at Policlinico Gemelli Foundation Hospital (until last year 2018), whose origins were mainly from Central and Southern Italy. This will give an overview of variant frequency in these geographic areas of Italy and provide data that could be used in clinical management of the patients.

## 2. Results

### 2.1. Results Next-Generation Sequencing

Among 2351 patients screened for *BRCA1/2* variants, 517 (22%) resulted carriers. The characteristics of this study group are shown in Table 1. All variants identified in both genes were analyzed, carefully examined, and classified according to several database including ENIGMA, ClinVar, LovD, and UMD and in reference to the literature. We found 249 individuals carrying a *BRCA1* variant, while 260 with a *BRCA2* one. Eight patients resulted in being carriers of a variant in both genes.

Results regarding *BRCA1* and *BRCA2* are shown in Table 2. The germline variants are located along the entire coding sequence of both genes. About the frequency of variants, we found that the number of *BRCA2* variants exceeded that of the *BRCA1* ones (180/517, 35% versus 102/517, 19.7%). Most of these PVs were distributed within exon 11 of each gene.

As reported in Table 3, within 26 probands (5.0%), including 14 (54%) BC and 11 (42%) OC, we identified a double or triple variant.

**Figure 1:**
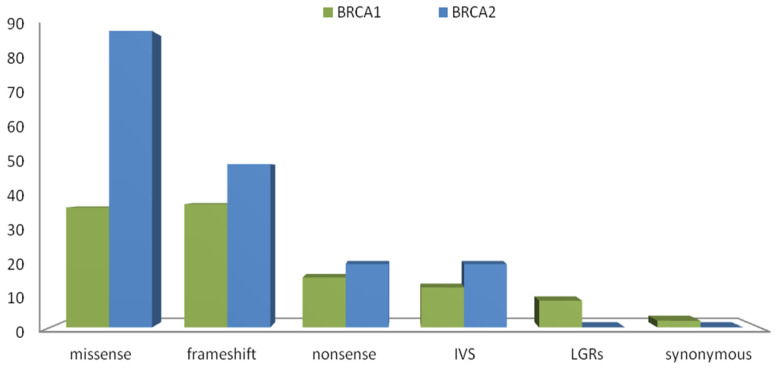
Variant spectrum in *BRCA1* and *BRCA2* genes. Histograms indicate the absolute number of all variants in *BRCA1* and *BRCA2,* identified in our population.

In detail, we observed the following variant types: frameshift (*n* = 37), missense (*n* = 36), nonsense (*n* = 15), intronic sequencing variants (*n* = 12), synonymous variants (*n* = 2) and large genomic rearrangements (*n* = 8) in *BRCA1*. Regarding the *BRCA2* gene, the variants identified were frameshift (*n* = 49), missense (*n* = 89), nonsense (*n* = 19), and intronic sequencing variants (*n* = 19) variants. Furthermore, no synonymous variations and no large genomic rearrangements in *BRCA2* (Figure 1) were detected. For both genes, most of the variants were classified as of Class 5 (67% in *BRCA1*, 47% in *BRCA2*). In addition, Class 3 (VUS) were 15% and 30% for *BRCA1* and *BRCA2,* respectively, while the remaining ones were classified, according to the reference database, such as conflict of pathogenicity (15% and 16% for *BRCA1* and *BRCA2,* respectively).

### 2.2. Novel Variants

**Figure 2:**
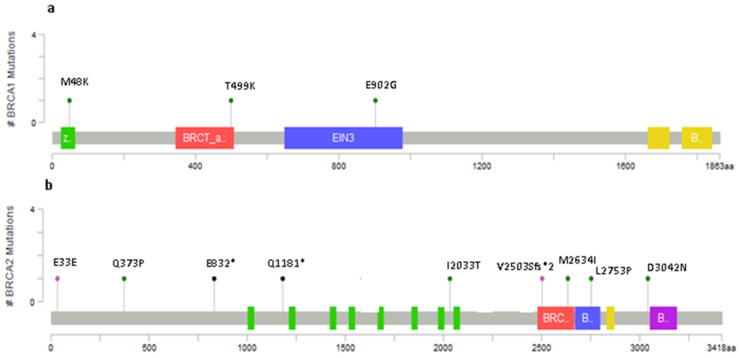
Lollipop plots showing the distribution of novel *BRCA1* variants (**a**) and *BRCA2* (**b**) identified in our patients’ group. The plots were obtained by the informatic tool Mutation Mapper—cBioPortal for Cancer Genomics (GenBank Reference *BRCA1*: NM_007300 and GenBank Reference *BRCA2*: NM_000059).

Although most of the variants reported were known, we indeed identified three novel alterations in *BRCA1* and nine in *BRCA2* which were not reported either from literature data or from the main reference databases. Apart from the c.143T>A and the c.4803dupT, found in two non-relative patients, all the remaining ones were identified only once (in bold in Table 2). The positions of the variants on *BRCA1* and *BRCA2* genes, excluding those predicted as “likely benign”, are plotted within Figure 2a,b, respectively.

The c.143T>A (p. Met48Lys, M48K) is a missense variant, reported only once in the literature by our previous paper [21] and now identified in two female patients with familial history of triple-negative breast cancer (TNBC) and OC developed at the age of 53 and 58 years, respectively. This variant is located in the amino-terminal RING domain, in a hot-spot region in which there are primarily clustered missense [23] and in which 57 variants out of 62 were classified as pathogenic. This variant was predicted as deleterious by VarSome and with moderate probability of pathogenicity by Priors. The computational verdict of pathogenicity was inferred by DANN, GERP, FATHMM, LRT, MutationTaster, FATHMM-MKL, and SIFT prediction, while only two predictions of benignity resulted from the Mutation Assessor and PROVEAN tools). Moreover, the Genomic Evolutionary Rate Profiling (GERP) score (4.01) and Align-GVGD score (C65) indicate that the Met amino acid in position 48 lies in a strongly conserved region further deposing in favor of a prediction of strong pathogenicity. The c.1496C>A was found in a female subject with OC at the age of 49 and caused the substitution of threonine with a lysine at the 499 position. It was defined with low pathogenicity by Priors (0.02 score), with a C0 Align-GVGD score. Although VarSome defined this variant as VUS, some evidences support a possible pathogenic role. In fact, the pathogenic computational verdict comes from predictions from DANN, GERP, FATHMM, Mutation Assessor, PROVEAN and SIFT (vs 3 benign predictions from LRT, MutationTaster and FATHMM-MKL). The c.2705A>G, p.(Glu902Gly), was detected in a 38 year old woman suffering from TNBC bilateral breast cancer. By analyzing this variant using Priors and Align-GVGD, the results indicated no pathogenic effect. VarSome analysis classified this variant as VUS because 6 out 9 prediction scores (i.e., GERP, FATHMM, Mutation Assessor, LRT, MutationTaster, and FATHMM-MKL) resulted as neutral/tolerated, while DANN, PROVEAN, and SIFT assigned to it a pathogenic effect. This was confirmed by a gnomAD exome frequency of 0.00000399 (0.00000882 in European non-Finnish) that is smaller than the 0.00001 threshold for *BRCA1* gene. This variant is located in *BRCA1* OCCR region. Among the 11 *BRCA2* novel variants, in silico analysis performed by VarSome, Priors, and Align-GVGD identified was as follows: 2 as likely benign, 5 VUS with low pathogenicity, and 4 as pathogenic variants (Table 2 and Table 3, respectively) since they introduced a stop codon change.

About *BRCA2* variants c.1118A>C and c.6098T>C, the in silico analysis predicted these as likely benign. In particular, we found a woman with OC at the age of 53 carrying in *BRCA2* with both these c.1118A>C variants coupled to the PV (c.658_659delGT). The c.1118A>C showed a Priors analysis score of 0.02, while VarSome gave a final Benign computational verdict coming from 6 benign predictions given by FATHMM, LRT, Mutation Assessor, Mutation Taster, PROVEAN, and FATHMM-MKL (against only three pathogenic predictions from DANN, GERP, and SIFT): we underline as this position is not conserved (GERP++ rejected substitutions = 5.6 is less than 8). A similar verdict was obtained for the c.6098T>C: this variant was found in a TNBC 39 year old woman. Regarding the c.6098T>C, VarSome analysis showed eight benign predictions from DANN, GERP, FATHMM, LRT, Mutation Assessor, Mutation Taster, PROVEAN, and FATHMM-MKL (versus no pathogenic predictions), since the position is not conserved (GERP++ rejected substitutions = −5.05 is less than 8). Furthermore, Priors analysis gave a score of 0.03.

Two novel frameshift and three novel nonsense variants can be considered as pathogenic (Class 5 according to ENIGMA guidelines, or at least Class 4), because the variant allele predicted to encode truncated, non-functional protein due to the creation of a new stop codon. In detail, regarding the novel PVs in the *BRCA2* gene: (a) no data are available about the frequency of c.3541C>T, located in OCCR region and reported in the dbSNP database (rs971408325) [24]; (b) the c.4803dupT in BRC REPEAT-RAD51 domain determines truncation; (c) the c.7506delC in the DNA-binding domain determines the frameshift effect with truncation of protein; (d) the c.2491_2492insT, creating a truncating site, is indeed located out of any regulative regions. For the other five novel *BRCA2* variants, the in silico analysis reported a verdict of VUS: in particular, c.99A>G found in a patient with BC and also carrier of c.9828A>T and c.517-4C>G variants, it was defined as VUS by VarSome database although the Priors and Align-GVGD scores (0.02 and C0, respectively) defined it as weak or a null probability of pathogenicity. The variants c.4769A>G and c.9124G>A, found in two healthy subjects belonging to families with history of BC, were defined VUS by VarSome software only because DANN and SIFT (0.9962 and 0.027 score, respectively) predicted them as pathogenic versus other tools that showed a prediction of benignity. In addition, Priors and Align-GVGD score, 0.02 and C0, respectively, corroborate this prediction. Regarding the c.7617+9_7617+12delTTGT, we obtained contrasting results since the VUS verdict was based on two different probabilities: (a) moderate pathogenicity, due to the absence of this variant in GnomAD exomes and genomes; (b) a benign computational verdict because one benign prediction from GERP versus no pathogenic predictions was obtained; (c) the position was not conserved (GERP++ rejected substitutions = 0.436 is less than 6.8).

Finally, the variant c.7902G>A was found concurrently with c.3635delA in a 67 year old OC patient. This variant is reported in the dbSNP database (rs1483170360) without any data regarding its clinical significance, while in GnomAD exomes, this allele showed a frequency of 4.224e-06 (very rare). Although VarSome defines this variant as a VUS, it should indeed be carefully considered, because there were eight pathogenic predictions from DANN, GERP, FATHMM, LRT, Mutation Assessor, MutationTaster, FATHMM-MKL, and SIFT ( versus 1 benign prediction from PROVEAN). Considering the family history and the identification of this novel *BRCA2* pathogenic variant, the genetic screening of first-degree relatives was strongly recommended. Genetic analysis performed on an affected maternal cousin (breast cancer at 48 years), resulted positive for both the c.7902G>A and the c.3635delA alterations, confirming the in cis status of these variants. Therefore, we can speculate as to this haplotype segregated with the disease in the two first-degree relatives, possibly supporting the eight scores of pathogenicity obtained in silico.

Finally, as reported within Table 3, 26 (5.1%) patients carried more than one variant (Class 5, 4 or 3): 24 carried two variants and two a combination of three (the latter involving only the *BRCA2* gene). In some cases (7/24; 29.2%) the concomitant presence of two Class 5 variants was detected: in 5/7 cases, the Class 5 double PV was present in *BRCA2* while in only two cases in both genes each one. Since patients n.22, 24, 25, and 26 did not report any history of Fanconi’s syndrome, we can hypothesize that the double PVs segregated in cis modality. 

### 2.3. Recurring Variants

The frequencies of recurring PVs are reported in Table 4 as referred to Human Genome Structural Variation Consortium (HGSV). The most frequent variants in *BRCA1* are c.5266dupC (9.6%) and c.4117G>T (9.2%), followed by other ones detected in a range between 1.6% to 5.2%. Among overall variants: nine were founder and, in particular, 6 were reported as Italian founders, and three of these had a high frequency: c.4117G>T (9.2%), c.4964_4982del (3.2%), and c.1360_1361delAG (1.6%) in *BRCA1.* Contrastingly, for *BRCA2,* the most frequent variants showed a homogeneous distribution spanning from 1.6% to 3.6%.

### 2.4. Variants Location and Type of Cancer

**Figure 3:**
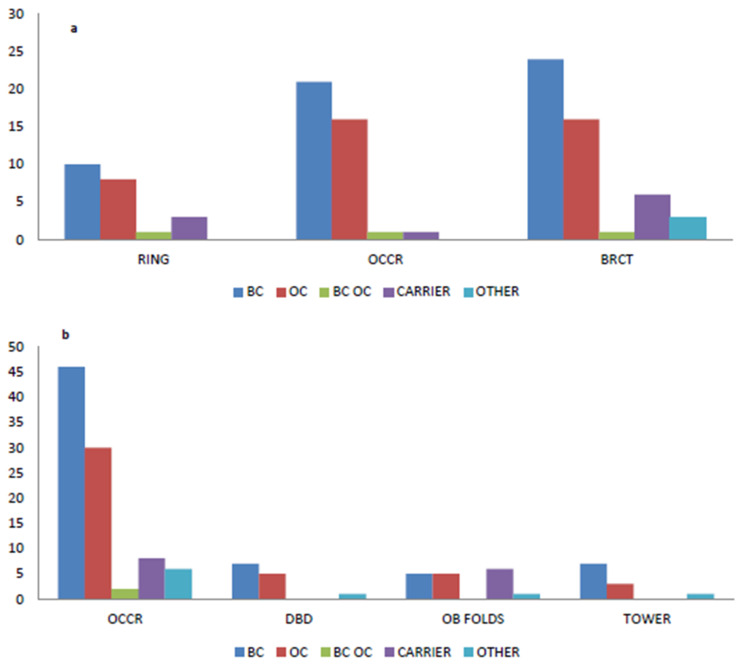
The histograms represent the absolute number of patients divided by BC, OC, BC/OC, carrier or other that show a variant located within one of the classic domains of the *BRCA1* (**a**) or *BRCA2* (**b**) genes.

By dividing our cohort into five subclasses (i.e., BC, OC, BC/OC, carriers, others), we were not able to evaluate a correlation between the type of PV and the specific patient’s disease, as most variants occur only once. In addition, we showed that there was no statistically significant correlation between *BRCA1* and/or *BRCA2* variants’ location and type of cancer (*p*-value 0.475 and 0.194 for *BRCA1* and *BRCA2*, respectively). In fact, in our cohort, 24 BC patients (44%) carried *BRCA1* variants falling into the BRCT domain while 16 OC patients (40%) showing variants in the OCCR and BRCT domains (Figure 3a); regarding *BRCA2,* 46 BC patients (70%) and 30 OC patients (71%) presented variants in the OCCR domain (Figure 3b). Moreover, no significant correlation among *BRCA1/2* variants and the putative regions identified by Rebbeck et al. [25] was found (*BRCA1* and *BRCA2* versus putative regions *p* = 0.558 and 0.362, respectively). From a clinical point of view, it is important to refer the outcome of a 78 year old triple-negative BC patient carrying two variants in the *BRCA1* gene: c.134+2T>C and c.2281G>C (patient number 7 in Table 3). This patient was treated with a modified Sikov regimen (reduced dose by age) for locally advanced triple-negative BC (cT2, cN2). Before the completion of treatment, she showed a local disease progression with breast enlargement, edema, and erythema as for inflammatory carcinoma. She performed CT scan that revealed systemic progressive disease with lung and bone metastases. Thereafter, the patient started olaparib with progressive disease after three months. We are waiting for somatic testing for TMB to understand what molecular changes have determined resistance to treatment.

## 3. Discussion

This study is the first report about the landscape of germline *BRCA1* and *BRCA2* PVs in a large cohort of patients affected by breast and ovarian cancer coming from the Central–South Italy. Most of the breast and ovary cancers are sporadic in nature, while only a small but significant percentage (5%–10% for BC and 20% for OC) can be referred to the hereditary or familial risk. The prevalence of *BRCA1/2* variants is different among both ethnic groups and geographical areas, showing a frequency between 17.6% to 29.8% in white European and Australian people while 9.4% to 21.7% in Asian countries. [26]. The rate of variants within Italian families with BC and/or OC cases is rather controversial and varies from 8% to 37%, according to different reports [21,27]. However, a large number of variants still has an unknown biological and clinical significance.

The present paper shows that the *BRCA1/2* variants are distributed along the entire sequence, with a higher frequency for *BRCA2* than *BRCA1*: in fact, we identified 103 *BRCA1* and 181 *BRCA2* deleterious variants. We underline that the most common variants are the founder variants *BRCA1* c.5266dupC and c.181T>G, which are characteristic of Ashkenazi Jews and Central Europe, respectively [28,29]. Furthermore, also the c.4117G>T (reported in a study of Di Giacomo et al. [30] as Abruzzo and Lazio founder) and the c.4964_4982del (which is a founder variant from the southern region of Calabria and Sicilia [16,31]) showed frequencies equal to 9.2% and 3.2%, respectively. Noteworthy, although the c.5123C>A was annotated by Diez et al. [32] as a Spain founder variant, it was still a quite frequent in Italy (5.2%): we can speculate that this high frequency could be due to the Spanish domination in the regions of Central–Southern Italy. About *BRCA2*, the distribution of recurring variants did not reflect any founder effect: finally, 89% (72/181) of *BRCA2* and 95% (94/106) of *BRCA1* variants can be considered rare or moderately rare because they detected in at least in 1 to 3 probands. As to the cases were two or three variants are present, no correlation was found between these and the early onset or the major recrudescence of the disease. About *BRCA1* c.*85A>G in UTR-3’ region, analysis by RegRNA 2.0 software did not identify the presence of any functional RNA motifs and sites in this position. Another aim of this study was to establish the possible correlation between the site of variant and phenotype effects. In 2015, Rebbeck et al. [25] explored the existence of portions of the gene with the relative variation in breast and ovarian cancer risk. Referring to this work, we tried to identify the areas for which the variant site could define a possible genotype/phenotypic effect related to BC/OC risk. Our data did not allow us to define new areas other than the already known as the OCCR or BCCR ones. This is probably due to the number of samples examined and, therefore, studies on larger groups would be needed to confirm the existence of these areas: nevertheless, we underline as this study was carried out on more than 2300 patients who undergone *BRCA1/2* genetic testing. One of the most interesting findings of the present study is the identification of novel variants exclusive of each patient, excluding only the c.143T>A that was reported in two women affected by triple-negative breast cancer (TNBC) and ovarian cancer with family history of hereditary cancer. The largest number (thirteen in total) of novel variants was detected in the *BRCA2* gene, while only six in the *BRCA1* gene, where three were predicted as pathogenic. In depth, the c.143T>A variant, dropping in the *BRCA1* RING domain, a very conserved region with a function as E3 ubiquitin ligase, could be speculated as affecting the ubiquitination process which is essential for tumor suppressor function of *BRCA1* protein. Moreover, for the c.1496C>A and c.2705A>G, identified in two females with OC and TNBC, respectively, there was a conflictual interpretation. The first is located out of any original OCCR region, but within the putative OCCR region (OCCRP) [27], while the second is in original OCCR region. Regarding these peculiar OCCR regions, although without any significantly statistical correlation, we underline that some patients with TNBC carried PVs falling into the OCCR or OCCRP regions (10/54 for *BRCA1* and 6/15 for *BRCA2*). Obviously, it would be necessary to study larger cohorts in order to validate our hypothesis. As *BRCA1*, also *BRCA2* plays an important role in tumor suppression and DNA repair process, probably for its direct interactions with Rad51 during homologous recombination in two different domains: the eight BRC repeat and DBD domain [33]. These interactions occur with *BRCA2*’s highly conserved residues, three oligonucleotide binding (OB) folds domains, that bind ssDNA: this mechanism supports the idea that *BRCA2* directly participates in recombinase-mediated strand exchange during DNA double-strand break (DSB) repair [34]. The two *BRCA2* (c.1118A>C and c.2491_2492insT) variants, are not located in any structural domain of particular interest. However, the c.2491_2492insT could have a damaging effect due to the creation of a truncated protein. The variants c.3541C>T and c.4803dupT can be considered as deleterious because they are located in the region of eight BRC repeats. Finally, the c.7506delC that causes the formation of a truncated protein in the most important site of *BRCA2*, being located in the DBD domain, the latter well conserved from *Arabidopsis* to humans. The importance of this domain in the overall function of gene, as a tumor suppressor, is due to the binding of *BRCA2* protein with Rad51 and their role in homologous recombination process. About novel variants for both genes classified as VUS (7/12), we believe that it is appropriate to carry out functional studies to assess their clinical role as soon as possible. Regarding patient number 7, who showed a resistance to treatments (including olaparib), we cannot exclude the presence of somatic *BRCA* reversal variant influencing the rate of drug response.

Finally, our findings can furtherly contribute to improve data regarding the frequency of such *BRCA1* and *BRCA2* variants in specific geographic area of Italy. Furthermore, the discovery of new *BRCA1/2* variants could allow us to identify new founder effects for some of these, in addition to those already described [30,35] and provide data useful for clinicians above all in order to plan personalized therapy and to extend a targeted screening in other family members.

## 4. Materials and Methods

### 4.1. Sample Collection

This is a retrospective single-center study performed at the Policlinico A. Gemelli Foundation in Rome. From January 2016 to 2018, a total of 2351 patients with BC and/or OC were referred to our Clinical Molecular Diagnostics Laboratory for genetic analysis of the *BRCA1* and *BRCA2* genes. Before assaying *BRCA1/2* genes, onco-genetic counselling was offered to all patients with early BC/OC onset cancer and/or contralateral BC and to healthy individuals belonging to high risk-families. Rules and criteria followed for genetic counseling and testing are reported in the Italian recommendations for Ovarian cancer provided by AIOM-SIGU-SIBioC-SIAPEC-IAP inter-society scientific Working Group [22] and following the National Comprehensive Cancer network [36] guidelines continuously updated until now. AIOM = Italian Society of Medical Oncology; SIGU = Italian Society of Human Genetics; SIBioC = Italian Society of Clinical Biochemistry and Clinical Molecular Biology; SIAPEC_IAP = Italian Society of Anatomic Pathology and Diagnostic Cytopathology. The present study matches with the Declaration of Helsinki, and the patients described regard those included within protocol approved by the Ethics Committee of Gemelli Hospital Foundation (Project ID: ESR14-10185, Approval date: 24/02/2016).

### 4.2. Molecular Testing

#### DNA Extraction and Next-Generation Sequencing (NGS) Pipeline

After informed consent was given, DNA was extracted from whole blood samples by Qiagen DNeasy Blood and Tissue kit on Qiacube instrument (Qiagen, Milan, Italy) and quantified successively by Qubit 3.0 Fluorimeter, according to the manufacturer’s instructions.

The *BRCA1* and *BRCA2* full gene screening was performed using DEVYSER BRCA NGS kit (DEVYSER, Hägersten, Sweden), according to the manufacturer’s instructions. Sequencing reaction was carried out on the Illumina MiSeq System (Illumina, San Diego, CA, USA) and data obtained were analyzed by CE- IVD Amplicom Suite Software v. 1.0 (SmartSeq, Novara, Italy) as already published [37]. All the called variants (including indels), when not recorded as polymorphisms, were confirmed, on a new DNA sample, extracted from a second aliquot of blood, by Sanger sequencing. The latter was performed using the BigDye Terminator Cycle Sequencing V3.1 on 3500 Genetic Analyzer (Rome, Italy) and analyzed by SeqScape3 software (Life Technology, Rome, Italy), according to the manufacturer’s instructions. The Amplicom Suite Software v.1.0, coupled to the Devycer chemistry, also provided data on Copy Number Variation (CNV) status: all CNV+ve results or those with a prediction of possible rearrangements, were reanalyzed by MAQ (Multiplex Amplicon Quantification, Genova, Italy) assay, as already published [38,39]. The *BRCA1* and *BRCA2* reference sequences were NG_005905.2, NM_007294.3 and NG_012772.3, NM_000059.3, respectively. The layout of this work complies with the Declaration of Helsinki ethical principles. 

All sequence variants were named according to Human Genome Variation Sequence systematic nomenclature [40]. ClinVar, LOVD v3.0, and Evidence-based Network for the Interpretation of Germline Mutant Alleles [10,41,42] databases were used as main reference. If the variants were not found in any of the databases including the 1000 Genome, dbSNP, ClinVar or HGMD [43], we defined them as novel. The impact of novel variants on protein function or structure was analyzed using VarSome [44] and UMD [45], an integrated search engine that allows to access multiple database, prediction tools and publications at a single site. Variant pathogenicity is reported using an automatic variant classifier that evaluates the submitted variant according to the ACMG guidelines [9], classifying it as one of “pathogenic”, “likely pathogenic”, ‘”likely benign”, “benign” or “uncertain significance”. Population frequency data are taken from Kaviar3 [46], gnomAD [47], ICGC Somatic [48]; pathogenicity predictions from dbNSFP [49], which compiles prediction scores from 20 different algorithms, and DANN [48]. Clinically relevant information (associated conditions, inheritance mode, publications, etc.) are retrieved from the CGD [50], and variants are also linked to any associated phenotypes in the Human Phenotype Ontology [50,51]. We used Align Grantham variation deviation analysis (ALIGN-GVGD) to identify missense changes of potential functional significance [51,52,53]. About *BRCA1* 3’UTR variant we performed the analysis using several software as microRNA.org, miRbase, microinspector, and RegRNA [54,55,56,57,58].

### 4.3. BRCA1 and BRCA2 Cluster Regions

**Figure 4:**
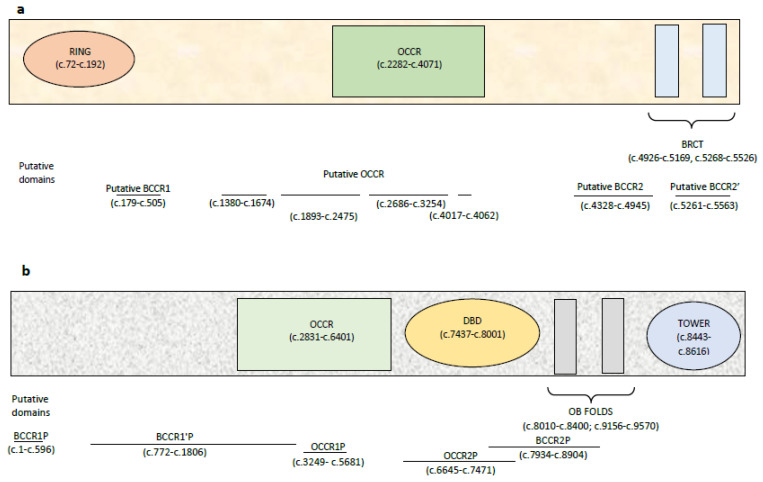
*BRCA1* and *BRCA2* cluster regions: (**a**) the classic regions of the *BRCA1* gene are shown within the rectangle, while the putative domains, identified by black lines, immediately below. The different putative regions are identified by black lines; (**b**) the classic regions of the *BRCA2* gene are shown within the rectangle, while the putative domains, identified by black lines, immediately below.

Next to the classic *BRCA1* and *BRCA2* cluster regions (OCCRs), Rebbeck et al. [25] described other cluster “putative BCCR regions” (BCCRP) in which it was observed a relative increase in breast cancer risk while a relative decrease in ovarian cancer risk for variants occurring in these and “putative OCCR regions” (OCCRP), with statistically significant evidence for a relatively higher ovarian cancer versus breast cancer risk for carriers of variant in these (Figure 4a,b).

### 4.4. Statistical Analysis

Descriptive statistical analysis was executed using SPSS version 23.0 software package (IBM Corporation, Armonk, NY, USA). Categorical variables were expressed as frequencies and percentages value. In order to compare categorical data, we used Pearson’s chi-squared test of association setting the limits of statistical significance as 0.05.

## 5. Conclusions

This study is the first report about the landscape of germline *BRCA1* and *BRCA2* PVs in a large cohort of patients affected by breast and ovarian cancer coming from the Central–South Italy, where, as recently published by Capoluongo et al. [59], it is quite probable to identify founder variants also in subjects belonging to apparently unrelated families. Moreover, as reported by Incorvaia et al. [60], in such regions, like Sicily, specific pattern of gene alteration can be identified in hereditary breast and ovarian cancers patients, where *BRCA1/2* PVs resulted as different from those usually detected in other geographical areas of Italy and Europe. Most of the breast and ovary cancers are sporadic in nature, while only a small but significant percentage (5–10% for BC and 20% for OC) can be referred to the hereditary or familial risk. The presence of *BRCA* PVs confer an elevated lifetime risk of developing breast and/or ovarian cancer. The early identifying PVs can be useful to help both the patients and their family members. The present paper shows that the *BRCA1/2* variants are distributed along the entire sequence, with a higher frequency for *BRCA2* than *BRCA1*: we identified 102 *BRCA1* and 180 *BRCA2* deleterious variants, 12 novel variants and recurring variants in both genes. The knowledge of BRCA status in individuals with BC and/or OC can help in treatment choosing, especially for OC and in planning a cost-effective screening among the first-degree relatives. This study reports in detail data regarding *BRCA1/2* significant variants identified in 517 out of 2351 BC and OC patients, providing an overview of variant frequency in a specific Italian geographic area: This information can be helpful in the clinical management of the patients. 

## Figures and Tables

**Table 1:** Prevalence of *BRCA1/2* variants in the 517 out of 2351 individuals screened.

Variables	Breast Cancer	Ovarian Cancer	Breast and Ovarian Cancer	Healthy Carriers	Other Cancer
Median Age	49 years	55 years	57 years	51 years	58 years
(range)	(28–83)	(29–85)	(40–79)	(22–86)	(32–79)
**Gender**					
Female	256	159	17	42	20
Male	7	-	-	15	2
**Variants Spectrum**					
*BRCA1* carriers (250)	119	85	9	28	9
*BRCA2* carriers (260)	140	70	8	29	13
*BRCA1/2* carriers (8)	4	4	-	-	-

**Table 2:** Spectrum of germline variants in *BRCA1* and *BRCA2* genes identified in 517 BC and OC patients.

***BRCA 1* Gene Variants**						
**Exon/Intron**	**HGVS Nucleotide**	**HGVS Protein**	**rs**	**Frequency**	**Variant Type**	**Class**
2	c.65T>C	p.(Leu22Ser)	rs80357438	2	M	5
IVS 2	c.80+1G>A	-	rs80358010	1	IVS	5
IVS 2	c.81-1G>C	-	rs80358018	2	IVS	5
3	c.134+2T>C	-	rs80358131	2	IVS	5
5	c.143T>A	p.(Met48Lys)	no rs	2	M	**Novel ^1,^°**
5	c.181T>G*	p.(Cys61Gly)	rs28897672	13	M	5
7	c.398G>A	p.(Arg133His)	rs80357357	3	M	CIP
IVS 7	c.441+5A>G	-	rs200358748	1	IVS	3
8	c.485_486delTG	p.(Val162GlufsTer19)	rs80357708	1	F	5
8	c.488G>C	p.(Arg163Thr)	rs1369043501	1	M	3
8	c.514delC	p.(Gln172AsnfsTer62)	rs80357872	5	F	5
IVS 8	c.547+2T>A	-	rs80358047	3	IVS	5
11	c.755G>A	p.(Arg252His)	rs80357138	2	M	CIP
11	c.798_799delTT	p.(Ser267LysfsTer19)	no rs	3	F	5
11	c.815_824dupAGCCATGTGG	p.(Thr276AlafsTer14)	rs387906563	1	F	5
11	c.843_846delCTCA	p.(Ser282TyrfsTer15)	rs80357919	1	F	5
11	c.850C>T	p.(Gln284Ter)	rs397509330	3	NS	5
11	c.946A>G	p.(Ser316Gly)	rs55874646	1	M	1
11	c.981_982delAT	p.(Cys328Ter)	rs80357772	1	F	5
11	c.997A>G	p.(Thr333Ala)	rs786201634	1	M	1
11	c.1016_1017insC	p.(Lys339AsnfsTer7)	rs1555592653	1	F	5
11	c.1063A>C	p.(Lys355Gln)	no rs	1	M	VUS
11	c.1081T>C	p.(Ser361Pro)	rs80356946	1	M	CIP
11	c.1217dupA	p.(Asn406LysfsTer6)	rs397508846	2	F	5
11	c.1252G>T	p.(Glu418Ter)	rs80357083	1	NS	5
11	c.1268C>T	p.(Ser423Phe)	no rs	1	M	**Novel^2,#^**
11	c.1297delG	p.(Ala433ProfsTer8)	rs80357794	1	F	5
11	c.1360_1361delAG*	p.(Ser454Ter)	rs80357969	4	F	5
11	c.1462dupA	p.(Thr488AsnfsTer2)	rs80357599	3	F	5
11	c.1496C>A	p.(Thr499Lys)	no rs	1	M	**Novel^#^**
11	c.1513A>T	p.(Lys505Ter)	rs397508877	1	NS	5
11	c.1612C>T	p.(Gln538Ter)	rs80356893	2	NS	5
11	c.1687C>T	p.(Gln563Ter)	rs80356898	3	NS	5
11	c.1703C>T	p.(Pro568Leu)	rs80356910	3	M	1
11	c.1895G>A	p.(Ser632Asn)	rs80356983	2	M	3
11	c.1953dupG	p.(Lys652GlufsTer21)	rs80357753	2	F	5
11	c.2037delGinsCC	p.(Lys679AsnfsTer4)	rs397508932	1	F	5
11	c.2077delGinsATA	p.(Asp693ThrfsTer8)	rs886039991	1	F	5
11	c.2195_2196delAAinsG	p.(Glu732GlyfsTer4)	rs397508948	1	F	5
11	c.2281G>C	p.(Glu761Gln)	rs397507198	2	M	3
11	c.2296_2297delAG	p.(Ser766Ter)	rs80357780	3	F	5
11	c.2405_2406delTG	p.(Val802GlufsTer7)	rs80357706	3	F	5
11	c.2501G>A	p.(Gly834Glu)	rs757383244	1	M	3
11	c.2518A>T	p.(Ser840Cys)	rs377475866	1	M	3
11	c.2529_2530delAA	p.(Ser844HisfsTer7)	rs886040046	1	F	5
11	c.2705A>G	p.(Glu902Gly)	no rs	1	M	**Novel^#^**
11	c.2760delA	p.(Gln921ArgfsTer79)	rs1064795769	1	F	5
11	c.3044dupG	p.(Asn1016LysfsTer2)	rs80357746	1	F	5
11	c.3082C>T	p.(Arg1028Cys)	rs80357049	1	M	1
11	c.3228_3229delAG *	p.(Gly1077AlafsTer8)	rs80357635	1	F	5
11	c.3285delA*	p.(Lys1095AsnfsTer14)	rs397509051	2	F	5
11	c.3331_3334delCAAG	p.(Gln1111AsnfsTer5)	rs80357701	1	F	5
11	c.3344_3346delAAG	p.(Glu1115del)	rs80358336	1	IFDEL	1
11	c.3454G>A	p.(Asp1152Asn)	rs80357175	1	M	CIP
11	c.3514G>T	p.(Glu1172Ter)	rs397509079	1	NS	5
11	c.3607C>T	p.(Arg1203Ter)	rs62625308	2	NS	5
11	c.3700_3704delGTAAA	p.(Val1234GlnfsTer8)	rs80357609	1	F	5
11	c.3756_3759delGTCT*	p.(Ser1253ArgfsTer10)	rs80357868	8	F	5
11	c.3868A>G	p.(Lys1290Glu)	rs80357254	1	M	3
11	c.3916_3917delTT	p.(Leu1306AspfsTer23)	rs80357678	1	F	5
11	c.3928dupA	p.(Thr1310AsnfsTer20)	rs886040176	1	F	5
11	c.3973delA	p.(Arg1325GlyfsTer11)	rs80357904	1	F	5
11	c.4054G>A	p.(Glu1352Lys)	rs80357202	1	M	3
11	c.4065_4068delTCAA	p.(Asn1355LysfsTer10)	rs80357508	1	F	5
IVS11	c.4096+1G>A	-	rs80358178	2	IVS	3
12	c.4117G>T*	p.(Glu1373Ter)	rs80357259	23	NS	5
12	c.4132G>A	p.(Val1378Ile)	rs28897690	3	M	1
12	c.4162C>T	p.(Gln1388Ter)	rs876660601	1	NS	5
12	c.4183C>T	p.(Gln1395Ter)	rs80357260	1	NS	5
13	c.4213A>G	p.(Ile1405Val)	rs80357353	1	M	CIP
13	c.4327C>T	p.(Arg1443Ter)	rs41293455	1	NS	5
13	c.4357insT	Ala1453ValfsX9/Ala1453GlnfsX3	no rs	2	F	5
14	c.4361T>C	p.(Val1454Ala)	rs587782606	1	M	CIP
14	c.4484G>T	p.(Arg1495Met)	rs80357389	3	M	5
IVS 14	c.4484+1G>T	-	rs80358063	1	IVS	5
IVS 15	c.4675+3A>G	-	rs80358082	1	IVS	3
16	c.4739C>T	p.(Ser1580Phe)	rs80357411	1	M	3
16	c.4882A>G	p.(Met1628Val)	rs80357465	1	M	CIP
16	c.4964_4982del*	p.(Ser1655TyrfsTer16)	rs80359876	8	F	5
17	c.5030_5033delCTAA	p.(Thr1677IlefsTer2)	rs80357580	3	F	5
17	c.5035_5039delCTAAT	p.(Leu1679TyrfsTer2)	rs80357623	1	F	5
17	c.5062_5064delGTT*	p.(Val1688del)	rs80358344	2	IFDEL	5
17	c.5073A>T	p.(Thr1691=)	no rs	5	S	5
IVS 17	c.5074+6C>G	-	rs80358032	1	IVS	1
18	c.5095C>T	p.(Arg1699Trp)	rs55770810	1	M	5
18	c.5106delA	p.(Lys1702AsnfsTer4)	rs80357553	1	F	5
18	c.5123C>A*	p.(Ala1708Glu)	rs28897696	13	M	5
18	c.5150delT	p.(Phe1717SerfsTer3)	rs80357720	1	F	5
20	c.5239C>T	p.(Gln1747Ter)	rs80357367	1	NS	5
20	c.5266dupC*	p.(Gln1756ProfsTer74)	rs397507247	24	F	5
21	c.5308G>T	p.(Gly1770Trp)	no rs	1	M	**Novel^2,^°**
21	c.5319dupC	p.(Asn1774GlnfsTer56)	rs80357823	1	F	5
22	c.5333A>G	p.(Asp1778Gly)	rs80357041	1	M	1/2
22	c.5353C>T	p.(Gln1785Ter)	rs80356969	4	NS	5
23	c.5431C>T	p.(Gln1811Ter)	rs397509283	1	NS	5
23	c.5434C>G	p.(Pro1812Ala)	rs1800751	1	M	4/5
23	c.5444G>A	p.(Trp1815Ter)	rs80356962	1	NS	5
IVS 23	c.5468-1G>A	-	rs80358048	1	IVS	5
24	c.5504G>C	p.(Arg1835Pro)	rs273902776	1	M	3
3-UTR	c.*85A>G		rs756518403	1	M	**Novel**
	c.(?_-1387-1)_(80+1_81-1)del	p.0?		3	LGR	5
	c.(212+1_213-1)_(441+1_442-1)del	p.?		1	LGR	5
	c.(4357+1_4358-1)_(4484+1_4485-1)del	p.?		1	LGR	5
	c.(4675+1_4676-1)_(5074+1_5075-1)del	p.?		1	LGR	5
	c.(5074+1_5075-1)_(5193+1_5192-1)del	p.?		4	LGR	5
	c.(5193+1_5194-1)_(5277+1_5278-1)del	p.?		1	LGR	5
	c.(5277+1_5277-1)_(5406+1_5407-1)del	p.?		2	LGR	5
***BRCA2* Gene Variants**						
**Exon/intron**	**HGVS Nucleotide**	**HGVS Protein**	**rs**	**Frequency**	**Variant Type**	**Class**
2	c.62A>G	p.(Lys21Arg)	rs397507367	2	M	3
IVS2	c.67+1G>A	-	rs81002796	3	IVS	5
3	c.289G>T	p.(Glu97Ter)	no rs	1	NS	5
4	c.353G>A	p.(Arg118His)	rs80358603	1	M	CIP
4	c.368_372delAAATG	p.(Lys123ArgfsTer5)	no rs	1	F	5
IVS 4	c.425+2T>C	-	rs876661045	1	IVS	4
IVS 6	c.516+1G>C	-	rs397507762	2	IVS	5
7	c.599C>T	p.(Thr200Ile)	rs587781402	1	M	3
IVS 7	c.632-2A>G	-	rs397507842	1	IVS	5
7	c.631G>A	p.(Val211Ile)	rs80358871	4	M	5
8	c.658_659delGT	p.(Val220IlefsTer4)	rs80359604	4	F	5
10	c.831T>G	p.(Asn277Lys)	rs28897705	1	M	CIP
10	c.1238delT	p.(Leu413HisfsTer17)	rs80359271	2	F	5
10	c.1244A>G	p.(His415Arg)	rs80358417	1	M	CIP
10	c.1247T>G	p.(Ile416Ser)	rs80358418	1	M	1/2
10	c.1257delT	p.(Cys419TrpfsTer11)	rs80359272	1	F	5
10	c.1259A>G	p.(Asp420Gly)	rs786201654	1	M	3
10	c.1296_1297delGA	p.8Asn433GlnfsTer18)	rs80359276	1	F	5
10	c.1322C>T	p.(Thr441Ile)	rs1064793062	1	M	3
10	c.1342C>T	p.(Arg448Cys)	rs80358422	1	M	CIP
10	c.1441A>G	p.(Ile481Val)	rs760559435	2	M	3
10	c.1514T>C	p.(Ile505Thr)	rs28897708	1	M	1
10	c.1550A>G	p.(Asn517Ser)	rs80358439	1	M	CIP
10	c.1670T>G	p.(Leu557Ter)	rs80358452	5	NS	5
10	c.1792A>G	p.(Thr598Ala)	rs28897710	1	M	1
10	c.1796_1800delCTTAT	p.(Ser599Ter)	rs276174813	3	NS	5
10	c.1813delA	p.(Ile605TyrfsTer9)	rs80359306	1	F	5
10	c.1820A>C	p.(Lys607Thr)	rs55962656	1	M	CIP
11	c.2014A>G	p.(Arg672Gly)	rs587781647	1	M	2
11	c.2094delA	p.(Gln699Serfs31)	rs80359323	1	F	5
11	c.2491_2492insT	p.(Glu832Ter)	no rs	1	NS	**Novel°** **(Class 4)**
11	c.2494G>T	p.(Glu832Ter)	rs786202875	1	NS	5
11	c.2651C>G	p.(Ser884Ter)	rs777421358	1	NS	5
11	c.2684delC	p.(Ala895ValfsTer9)	rs80359342	1	F	5
11	c.2808_2811delACAA	p.(Ala938ProfsTer21)	rs80359351	4	F	5
11	c.2821G>A	p.(Val941Met)	rs863224586	1	M	3
11	c.2836delG	p.(Asp946IlefsTer14)	rs80359358	1	F	5
11	c.2905C>T	p.(Gln969Ter)	rs886038080	1	NS	5
11	c.2944A>C	p.(Ile982Leu)	rs28897717	1	M	CIP
11	c.3443A>G	p.(Gln1148Arg)	rs200808363	1	M	3
11	c.3499A>G	p.(Ile1167Val)	rs276174834	1	M	3
11	c.3541C>T	p.(Gln1181Ter)	no rs	1	NS	**Novel°** **(Class 4)**
11	c.3551G>C	p.(Gly1184Ala)	rs431825309	1	M	3
11	c.3635delA	p.(Asn1212Metfs16)	no rs	1	F	5
11	c.3680_3681delTG	p.(Leu1227GlnfsTer5)	rs80359395	2	F	5
11	c.3683A>G	p.(Asn1228Ser)	rs786202838	1	M	3
11	c.3723T>G	p.(Phe1241Leu)	rs587782723	1	M	3
11	c.3744_3747delTGAG	p.(Ser1248ArgfsTer10)	rs80359403	1	F	5
11	c.3847_3848delGT	p.(Val1283LysfsTer2)	rs80359405	1	F	5
11	c.3860delA	p.(Asn1287IlefsTer6)	rs80359406	1	F	5
11	c.3962A>G	p.(Asp1321Gly)	rs80358645	1	M	2
11	c.4131_4132insTGAGGA	p.(Thr1378Ter)	rs80359429	6	IFINS	5
11	c.4133_4136delCTCA	p.(Thr1378ArgfsTer9)	rs80359430	2	F	5
11	c.4284dupT	p.(Gln1429SerfsTer9)	rs80359439	4	F	5
11	c.4285_4286insT	p.(Gln1429LeufsTer9)	rs886040518	1	F	5
11	c.4325C>A	p.(Ser1442Ter)	rs80358670	1	NS	5
11	c.4334A>C	p.(Lys1445Thr)	no rs	1	M	3
						
11	c.4419delC	p.(Asn1473LysfsTer6)	rs1064794337	1	F	5
11	c.4574A>G	p.(His1525Arg)	rs397507336	1	M	3
11	c.4647_4650delAGAG	p.(Lys1549AsnfsTer18)	rs397507734	1	F	5
11	c.4769A>G	p.(Lys1590Arg)	no rs	1	M	**Novel^1, #^**
11	c.4803dupT	p.(Lys1602Ter)	no rs	1	NS	**Novel^1,^°** **(Class 4)**
11	c.4899_4902delCTTT	p.(Phe1634Ter)	no rs	1	F	**Novel^2,^°** **(Class 4)**
11	c.4936_4939delGAAA	p.(Glu1646GlnfsTer23)	rs80359473	1	F	5
11	c.5073dupA	p.(Trp1692MetfsTer3)	rs80359479	3	F	5
11	c.5158dupT	p.(Ser1720PhefsTer7)	rs80359489	2	F	5
11	c.5224_5229delAACAGT	p.(Asn1742_Ser1743del)	rs276174855	1	IFDEL	3
11	c.5239_5240insT	p.(Asn1747IlefsTer8)	rs80359500	1	F	5
11	c.5261A>G	p.(Asp1754Gly)	rs772772727	1	M	3
11	c.5345A>C	p.(Gln1782Pro)	rs758959174	1	M	3
11	c.5351_5352dupA	p.(Asn1784LysfsTer3)	rs80359507	4	F	5
11	c.5423T>C	p.(Ile1808Thr)	rs397507350	1	M	CIP
11	c.5428G>A	p.(Val1810Ile)	rs80358766	1	M	3
11	c.5492T>C	p.(Ile1831Thr)	rs587782007	1	M	3
11	c.5634C>G	p.(Asn1878Lys)	rs80358784	1	M	1
11	c.5722_5723delCT	p.(Leu1908ArgfsTer2)	rs80359530	2	F	5
11	c.5796_5797delTA	p.(His1932GlnfsTer12)	rs80359537	2	F	5
11	c.5851_5854delAGTT	p.(Ser1951TrpfsTer11)	rs80359543	1	F	5
11	c.5885T>C	p.(Ile1962Thr)	rs1060502377	1	M	CIP
11	c.5897A>G	p.(His1966Arg)	rs80358823	1	M	CIP
11	c.5946delT	p.(Ser1982ArgfsTer22)	rs80359550	2	F	5
11	c.5959C>T	p.(Gln1987Ter)	rs80358828	1	NS	5
11	c.5971G>A	p.(Ala1991Thr)	no rs	1	M	**Novel^2, $^**
11	c.5986G>A	p.(Ala1996Thr)	rs80358833	1	M	CIP
11	c.6024dupG	p.(Gln2009AlafsTer9)	rs80359554	1	F	5
11	c.6037A>T	p.(Lys2013Ter)	rs80358840	4	NS	5
11	c.6037A>G	p.(Lys2013Glu)	rs80358840	1	M	3
11	c.6039delA	p.(Val2014TyrfsTer26)	rs876660637	2	F	5
11	c.6078_6079delAA	p.(Glu2028ArgfsTer20)	rs80359557	1	F	5
11	c.6098T>C	p.(Ile2033Thr)	no rs	1	M	**Novel^$^**
11	c.6131G>C	p.(Gly2044Ala)	rs56191579	1	M	CIP
11	c.6267_6269delGCAinsC	p.(Glu2089AspfsTer2)	rs276174868	1	F	5
11	c.6322C>T	p.(Arg2108Cys)	rs55794205	1	M	1
11	c.6405_6409delCTTAA	p.(Asn2135LysfsTer3)	rs80359584	1	F	5
11	c.6468_6469delTC	p.(Gln2157IlefsTer18)	rs80359596	4	F	5
11	c.6486_6489delACAA	p.(Lys2162AsnfsTer5)	rs80359598	2	F	5
11	c.6496G>T	p.(Val2166Leu)	rs750084851	1	M	3
11	c.6590_6591insA	p.(Glu2198Ter)	no rs	1	F	**Novel^2,^°** **(Class 4)**
11	c.6591_6592delTG	p.(Glu2198AsnfsTer4)	rs80359605	9	F	5
11	c.6650A>G	p.(Lys2217Arg)	rs1555284781	1	M	3
11	c.6761_6762delTT	p.(Phe2254TyrfsTer6)	rs80359624	1	F	5
IVS 11	c.6841+1G>T	-		1	IVS	3
12	c.6875A>G	p.(Glu2292Gly)	rs397507378	1	M	3
13	c.7007G>A	p.(Arg2336His)	rs28897743	7	M	5
13	c.7007G>C	p.(Arg2336Pro)	rs28897743	4	M	5
IVS13	c.7007+5G>A	-	rs81002816	1	IVS	3
14	c.7057G>C	p.(Gly2353Arg)	rs80358935	1	M	1
14	c.7072T>C	p.(Ser2358Pro)	rs80358937	1	M	3
14	c.7180A>T	p.(Arg2394Ter)	rs80358946	1	NS	5
14	c.7225C>T	p.(Pro2409Ser)	no rs	1	M	**Novel^2, #^**
14	c.7435+10G>A	-	rs81002793	1	IVS	CIP
15	c.7505G>A	p.(Arg2502His)	rs56070345	1	M	1
15	c.7506delC	p.(Val2503SerfsTer21)	no rs	1	F	**Novel°** **(Class 4)**
15	c.7561delA	p.(Ile2521SerfsTer3)	no rs	1	F	5
15	c.7617+9_7617+12delTTGT	-	no rs	1	IVS	**Novel^#^**
16	c.7636T>C	p.(Ser2546Pro)	rs1555286392	1	M	3
IVS 16	c.7806-2A>G	-	rs81002836	1	IVS	5
17	c.7857G>A	p.(Trp2619Ter)	rs80359011	2	NS	5
17	c.7878G>C	p.(Trp2626Cys)	rs80359013	1	M	5
18	c.7994A>G	p.(Asp2665Gly)	rs28897745	2	M	1
18	c.8009C>T	p.(Ser2670Leu)	rs80359035	1	M	CIP
18	c.8245C>T	p.(Gln2749Ter)	rs1135401925	2	NS	5
18	c.8249_8251delAGA	p.(Lys2750del)	rs80359703	1	IFDEL	3
18	c.8258T>C	p.(Leu2753Pro)	rs786203357	1	M	**Novel^2,#^**	
18	c.8299C>T	p.(Pro2767Ser)	rs587782619	2	M	3
19	c.8478C>A	p.(Tyr2826Ter)	rs776353983	2	NS	5
IVS 19	c.8487+1G>A	-	rs81002798	6	IVS	5
20	c.8537_8538delAG*	p.(Glu2846GlyfsTer22)	rs80359714	1	F	5
20	c.8567A>C	p.(Glu2856Ala)	rs11571747	1	M	CIP
IVS 20	c.8632+5A>G	-	rs763224070	1	IVS	2
IVS 21	c.8754+4A>G	-	rs81002893	2	IVS	5
IVS 21	c.8755-1G>A	-	rs81002812	4	IVS	5
22	c.8878C>T	p.(Gln2960Ter)	rs80359140	2	NS	5
22	c.8915T>G	p.(Leu2972Trp)	rs80359142	1	M	3
IVS 22	c.8954-1_8955delGTTinsAA	-	rs276174916	1	IVS	5
23	c.9097delA	p.(Thr3033LeufsTer29)	rs397507419	3	F	5
23	c.9104A>C	p.(Tyr3035Ser)	rs80359165	1	M	CIP
23	c.9116C>T	p.(Pro3039Leu)	rs80359167	2	M	CIP
24	c.9124G>A	p.(Asp3042Asn)	no rs	1	M	**Novel^#^**
24	c.9148C>T	p.(Gln3050Ter)	rs80359170	1	NS	5
24	c.9154C>T	p.(Arg3052Trp)	rs45580035	1	M	5
24	c.9171C>G	p.(Phe3057Leu)	rs747615055	1	M	3
25	c.9271G>A	p.(Val3091Ile)	rs80359194	1	M	CIP
25	c.9275A>G	p.(Tyr3092Cys)	rs80359195	1	M	CIP
25	c.9364G>A	p.(Ala3122Thr)	rs587782313	1	M	CIP
25	c.9382C>T	p.(Arg3128Ter)	rs80359212	2	NS	5
25	c.9413dupT	p.(Leu3138PhefsTer12)	rs876659435	1	F	5
IVS 25	c.9501+3A>T	-	rs61757642	2	IVS	1
IVS 25	c.9502-12T>G	-	rs81002803	1	IVS	1
26	c.9581C>A	p.(Pro3194Gln)	rs28897760	2	M	CIP
26	c.9583A>G	p.(Thr3195Ala)	rs80359227	1	M	CIP
26	c.9613_9614delGCinsCT	p.(Ala3205Leu)	rs276174926	3	M	3
27	c.9676delT	p.(Tyr3226IlefsTer23)	rs80359774	3	F	5
27	c.9959_9961delCTC	p.(Pro3320del)	rs745685382	2	IFDEL	3
27	c.10024G>A	p.(Glu3342Lys)	rs28897761	1	M	CIP
27	c.10040T>C	p.(Ile3347Thr)	rs587782373	1	M	3

rs: reference sequence; M: missense; NS: nonsense; F: frameshift; IVS: intronic sequencing variants; LGR: large genomic rearrangement; IFDEL: inframe deletion; IFINS: inframe insertion; 5: pathogenic variant; 4: likely pathogenic variant; this classification was also attributed to those novel variants with a canonical deleterious effect (terminator or frameshift); 3: variant unknown or of uncertain significance; CIP: conflicting interpretations of pathogenicity (generally referred to variants temporarily classified in ClinVar database as of uncertain significance and likely benign/benign) c. * founder. In silico analysis data. ° Pathogenic/likely pathogenic. # VUS. $ benign/likely benign; 1 = see Reference [19]; 2 = see Reference [22].

**Table 3:** Combination of Class 5, Class 4, and Class 3 variants in the *BRCA1* and *BRCA2* genes within each patient.

Patients with Double or Triple Gene Variants							
List of Patient	BC/OC	Gene	HGVS Nucleotide	HGVS Protein	rs	Variant Type	Class
1	BC	*BRCA1*	c.3228_3229delAG;	p.(Gly1077AlafsTer8)	no rs	F	5
		*BRCA2*	c.464G>C	p.(Arg155Thr)	rs377639990	M	3
2	BC	*BRCA2*	c.1238delT	p.(Leu413HisfsTer17)	rs80359271	F	5
		*BRCA1*	c.5095C>T	p.(Arg1699Trp)	rs55770810	M	5
3	BC	*BRCA1*	c.798_799delTT	p.(Ser267LysfsTer19)	no rs	F	5
		*BRCA2*	c.6290C>T	p.(Thr2097Met)	rs80358866	M	1
4	OC	*BRCA1*	c.5062_5064delGTT	p.(Val1688del)	rs80358344	IFD	5
		*BRCA2*	c.4054G>T	p.(Asp1352Tyr)	rs80358655	M	3
5	OC	*BRCA2*	c.800G>A	p.(Gly267Glu)	rs80359036	M	CIP
		*BRCA1*	c.4213A>G	p.(Ile1405Val)	rs80357353	M	CIP
6	OC	*BRCA2*	c.5796_5797delTA	p.(His1932GlnfsTer12)	rs80359537	F	5
		*BRCA1*	c.(?_-232)_(4096+1_4097-1)del	p.0?	-	LGR	5
7	BC	*BRCA1*	c.134+2T>C	-	rs80358131	IVS	5
		*BRCA1*	c.2281G>C	p.(Glu761Gln)	rs397507198	M	3
8	BC	*BRCA1*	c.3756_3759delGTCT	p.(Ser1253ArgfsTer10)	rs80357868	F	5
		*BRCA2*	c.3381delT	p.(Phe1127LeufsTer23)	no rs	F	3
9	OC	*BRCA1*	c.4964_4982del	p.(Ser1655TyrfsTer16)	rs80359876	F	5
		*BRCA1*	c.525G>A	p.(Lys175=)	rs1555594837	S	3
10	OC	*BRCA2*	c.2808_2811delACAA	p.(Ala938ProfsTer21)	rs80359351	F	5
		*BRCA2*	c.9116C>T	p.(Pro3039Leu)	rs80359167	M	CIP
11	BC	*BRCA2*	c.2808_2811delACAA	p.(Ala938ProfsTer21)	rs80359351	F	5
		*BRCA2*	c.9116C>T	p.(Pro3039Leu)	rs80359167	M	CIP
12	OC	*BRCA2*	c.865A>G	p.(Asn289Asp)	rs766173	M	CIP
		*BRCA2*	c. 5126A>C	p.(Asp1709Ala)	rs786202836	M	3
13	BC	*BRCA2*	c.99A>G	p.(Glu33=)	no rs	S	**Novel^#^**
		*BRCA2*	c.9828A>T	p.(Arg3276Ser)	rs80359245	M	3
		*BRCA2*	c.517-4C>G	-	rs81002804	IVS	3
14	OC	*BRCA2*	c.658_659delGT	p.(Val220IlefsTer4)	rs80359604	F	5
		*BRCA2*	c.1118A>C	p.(Gln373Pro)	no rs	M	**Novel^$^**
15	OC	*BRCA2*	c.3635delA	p.(Asn1212Metfs16)	no rs	F	5
		*BRCA2*	c.7902G>A	p.(Met2634Ile)		M	**Novel^#^**
16	Other	*BRCA2*	c.8452G>A	p.(Val2818Ile)	rs80359094	M	3
		*BRCA2*	c.191C>T	p.(Thr64Ile)	rs397507615	M	3
17	BC	*BRCA2*	c.5959C>T	p.(Gln1987Ter)	rs80358828	NS	5
		*BRCA2*	c.9038C>T	p.(Thr3013Ile)	rs28897755	M	1
18	BC	*BRCA2*	c.7008-2A>T	-	rs81002823	IVS	5
		*BRCA2*	c.631G>A	p.(Val211Ile)	rs80358871	M	5
19	OC	*BRCA1*	c.5266dupC	p.(Gln1756ProfsTer74)	rs397507247	F	5
		*BRCA2*	c.8262T>G	p.(His2754Gln)	rs587776472	M	3
20	BC	*BRCA2*	c.5202A>C	p.(Glu1734Asp)	rs1243093278	M	3
		*BRCA2*	c.5867A>T	p.(Asp1956Val)	rs1309562690	M	3
21	BC	*BRCA2*	c.2049_2050delTC	p.(Ser683Argfs)	rs80359319	F	5
		*BRCA2*	c.5191C>T	p.(His1731Tyr)	rs80358745	M	3
22	OC	*BRCA2*	c.631G>A	p.(Val211Ile)	rs80358871	M	5
		*BRCA2*	c.7008-2A>T	-	rs81002823	IVS	5
		*BRCA2*	c.79A>G	p.(Ile27Val)	rs80359034	M	3
23	BC	*BRCA2*	c.8567A>C	p.(Glu2856Ala)	rs11571747	M	CIP
		*BRCA2*	c.7008-62A>G	-	rs76584943	IVS	CIP
24	BC	*BRCA2*	c.631G>A	p.(Val211Ile)	rs80358871	M	5
		*BRCA2*	c.7008-2A>T	-	rs81002823	IVS	5
25	BC	*BRCA2*	c.2905C>T	p.(Gln969Ter)	rs886038080	NS	5
		*BRCA2*	c.6447_6448dupTA	p.(Lys2150IlefsTer19)	rs397507858	F	5
26	OC	*BRCA2*	c.631G>A	p.(Val211Ile)	rs80358871	M	5
		*BRCA2*	c.7008-2A>T	-	rs81002823	IVS	5

**Table 4:** Recurring pathogenic variants among BC and OC patients.

Gene	HGVS Nucleotide	HGVS Protein	*N* (%)
***BRCA1***	c.5266dupC	p.Gln1756Profs	24 (9.6)
	c.4117G>T	p.Glu1373Ter	23 (9.2)
	181T>G	p.Cys61Gly	13 (5.2)
	c.5123C>A	p.Ala1708Glu	13 (5.2)
	c.3756_3759delGTCT	p.Ser1253fs	8 (3.2)
	c.4964_4982del	p.Ser1655fs	8 (3.2)
	c.514delC	p.Gln172Asnfs	5 (2)
	c.1360_1361delAG	p.Ser454Ter	4 (1.6)
	DEL EXONS 18-19		4 (1.6)
***BRCA2***	c.6591_6592delTG	p.Glu2198fs	9 (3.6)
	c.7007G>A	p.Arg2336His	7 (2.8)
	c.4131_4132insTGAGGA	p.Thr1378Ter	6 (2.4)
	c.8487+1G>A	IVS19+1G>A	6 (2.4)
	c.631G>A	p.Val211Ile	4 (1.6)
	c.7008-2A>T	IVS13-2A>T	4 (1.6)
	c.658_659delGT	p.Val220fs	4 (1.6)
	c.2808_2811delACAA	p.Ala938Profs	4 (1.6)
	c.4284dupT	p.Gln1429fs	4 (1.6)
	c.5351_5352dupA	p.Asn1784Lysfs	4 (1.6)
	c.6037A>T	p.Lys2013Ter	4 (1.6)
	c.6468_6469delTC	p.Gln2157fs	4 (1.6)
	c.7007G>C	p.Arg2336Pro	4 (1.6)
	c.8755-1G>A	IVS21-1G>A	4 (1.6)
